# A structured framework for improving outbreak investigation audits

**DOI:** 10.1186/1471-2458-9-472

**Published:** 2009-12-18

**Authors:** Craig B Dalton, Tony D Merritt, David N Durrheim, Sally A Munnoch, Martyn D Kirk

**Affiliations:** 1 Hunter New England Population Health, Longworth Ave, Wallsend, 2287, NSW, Australia; 2 Conjoint academic with The University of Newcastle, Australia; 3 Office of Health Protection, Commonwealth Department of Health and Ageing, Woden, 2606, ACT, Australia; 4 National Centre for Epidemiology & Population Health, The Australian National University, Canberra, 0200ACT, Australia

## Abstract

**Background:**

Outbreak investigation is a core function of public health agencies. Suboptimal outbreak investigation endangers both public health and agency reputations. While audits of clinical medical and nursing practice are conducted as part of continuous quality improvement, public health agencies rarely make systematic use of structured audits to ensure best practice for outbreak responses, and there is limited guidance or policy to guide outbreak audit.

**Methods:**

A framework for prioritising which outbreak investigations to audit, an approach for conducting a successful audit, and a template for audit trigger questions was developed and trialled in four foodborne outbreaks and a respiratory disease outbreak in Australia.

**Results:**

The following issues were identified across several structured audits: the need for clear definitions of roles and responsibilities both within and between agencies, improved communication between agencies and with external stakeholders involved in outbreaks, and the need for development of performance standards in outbreak investigations - particularly in relation to timeliness of response. Participants considered the audit process and methodology to be clear, useful, and non-threatening. Most audits can be conducted within two to three hours, however, some participants felt this limited the scope of the audit.

**Conclusion:**

The framework was acceptable to participants, provided an opportunity for clarifying perceptions and enhancing partnership approaches, and provided useful recommendations for approaching future outbreaks. Future challenges include incorporating feedback from broader stakeholder groups, for example those of affected cases, institutions and businesses; assessing the quality of a specific audit; developing training for both participants and facilitators; and building a central capacity to support jurisdictions embarking on an audit. The incorporation of measurable performance criteria or sharing of benchmark performance criteria will assist in the standardisation of outbreak investigation audit and further quality improvement.

## Background

Outbreak investigation is a core function of public health agencies. Suboptimal outbreak investigation endangers both public health and agency reputations. Surprisingly, there is little guidance on enhancing the quality of outbreak investigation and control provided to public health agencies. Audits of clinical medical and nursing practice are conducted as part of continuous quality improvement and particularly where significant events have occurred, such as unexpected deaths[1]. They are sometimes referred to as "clinical audits", "morbidity and mortality meetings", "critical event auditing" or "facilitated case discussions". Such audits are undertaken to identify ways of improving practice by identifying barriers to best practice, highlighting exemplary practice, and for debriefing staff after a particularly stressful incident.

While recommendations for practice improvement in outbreak investigation may be found in the discussion section of journal articles reporting on outbreak responses and published reports may critique limited aspects of outbreak response performance, a comprehensive review of outbreak investigation practice often only follows high profile events subjected to an independent government audit, or a coronial inquiry[2-5]. Public health agencies should make more systematic use of structured audits to ensure best practice for all outbreak responses, but there is limited guidance or policy development for outbreak audit[6].

A framework for prioritising which outbreak investigations to audit and how to conduct an audit of outbreak investigation practice is presented. The application of this approach in four foodborne outbreaks and a respiratory disease outbreak in an Australian context is reviewed.

## Methods

The audit methodology has evolved since its initial trial during a national workshop to test outbreak response guidelines in 1997 which focused primarily on the rationale, selection of outbreaks, and development and use of the Audit Trigger Questions 1 in the context of a multi-state outbreak[7,8]. It has been refined and trialled more extensively since 2005 when dispute resolution principles were integrated into the methods - not because the process inherently involves conflict but to prevent the development of conflict and create an environment in which the mutual interests of all parties can best be addressed[9,10].

The success of a structured audit is dependent upon the appropriate:

• Selection of outbreaks to be audited

• Engagement and preparation of stakeholders

• Process of the audit

• Confidentiality agreement, where necessary

• Implementation and dissemination of recommendations

### Selection of outbreaks to be audited

Public health activities should be evaluated as part of quality assurance and outbreaks almost always hold lessons for service improvement. However, some outbreaks, characterised by criteria in Table 1, may be worthy of more comprehensive and detailed audit. In our experience outbreaks should be audited within six months of the resolution of the outbreak to optimise recollection of events.

**Table 1:** Criteria for selecting outbreaks for audit.

Criteria	Example/Details
Outbreak of local, state, national or international significance	Multi-state outbreaks Disease reported internationally Exotic/emerging diseases Highly virulent pathogens in terms of death/hospitalisation or high attack rate Tourist facilities National coordination required

Unusual outbreaks	Large outbreaks New epidemiological or laboratory methods Complex epidemiological investigations Special learning opportunities, eg rare pathogen or unusual mode of transmission

System issues	Timeliness of epidemiological, environmental or laboratory response Demonstrated failure of routine public health practice Perceived failure of health protection standards or protocols Inter-jurisdictional communication challenges Complex outbreak coordination Cultural differences between jurisdictions/agencies Stress among investigating team members

Legal or Administrative	Litigation or administrative review of decision making

Public/Media concern	A high degree of community concern about the outbreak. Confidentiality provisions should be considered.

A benefit of normalising structured audit as part of routine practice is that audits associated with inter-jurisdictional conflict will be less threatening to stakeholders if the process is regarded as routine. It is important that audits not only be conducted following perceived system "failures" but also to identify and promote good practice. Ideally, agencies responsible for outbreak investigation should audit at least one or two investigations annually, even where specific selection criteria are not satisfied, as there can be significant learning from small or "routine" investigations.

While there are costs involved in conducting a structured audit, proactively addressing communication and system performance issues may well be cost saving from an organisational perspective, however, awareness of the opportunity costs involved in conducting an audit should influence the careful selection of outbreaks to be audited and the efficient conduct of the audit. We believe most structured audits can be conducted within two to three hours and should be restricted to this time limit.

### Engagement and preparation of stakeholders

It is essential that the focus of the audit is on improving future practice rather than on laying blame or identifying individual people or agencies for criticism. Participants may have endured a stressful experience during the outbreak period and extensive media scrutiny. In some outbreak investigations, legal and media scrutiny can lead to criticism of key personnel investigating outbreaks. Staff may be sensitive to an audit of their work. It is important that all participants come to the audit with the expectation of a positive outcome that will improve future practice, rather than fearing further criticism.

Participants should be drawn from the pool of stakeholders who can assist in the process of the audit or those who's future practice will benefit from participation including both higher level managers and frontline staff. Including participants from external but collaborative agencies will bring more divergent viewpoints to the audit and extend the range of issues explored and resolutions available. We have conducted audits with up to 20 participants, however, the number of participants should be balanced with the scope of the audit, the issues to be reviewed and the time available.

Generally, it is preferable to use a skilled facilitator. Using an external facilitator has the advantage of independence and bringing a fresh perspective. However, an external facilitator may not know the roles of key people and agencies involved in the outbreak response. A facilitator should have some experience of outbreak investigation but their key expertise should be in the process of facilitation. The facilitator should ensure constructive framing of discussion and reorient interpersonal conflict to address system issues if possible through interest-based negotiation that focuses on the underlying interest of the parties rather than their competing claims or positions[10].

The facilitator is responsible for 1) explaining the aims, ground rules, and principles of the audit, 2) maintaining the structure of the audit, 3) facilitating the process including seeking agreement on key themes and scope of the audit, encouraging contributions broadly across participants, managing time, clarifying and summarising issues, clarifying assumptions, 4) maintaining an impartial perspective, 5) summarising the outcome of the audit and assisting in writing a report, and 6) checking on progress of actions approximately six weeks after the audit.

### Process of the audit

#### Preparing for the Structured Audit

The lead agency in the investigation will usually call for an audit of an investigation and define the expectations of the audit outcome at the outset. The terms of reference, the scope of the audit, attendees, duration and the expected product should be defined in consultation with participants and in advance so that participants are supportive and prepared for the meeting. The major objective of the audit should be framed as a neutral system performance statement or question.

We use the term "structured" to describe two aspects of this methodology - first to denote the structure used in the Audit Trigger Questions to ensure a comprehensive range of issues are addressed and secondly to denote the structure for audit meeting preparation and conduct. The Audit Trigger Questions (Additional file 1) may be used to suggest specific areas for review. Since an outbreak is a public health emergency, the four categories of prevention/mitigation, preparedness, response, and recovery from emergency management theory were used to frame the Audit Trigger Questions to encourage the entire spectrum of response to be considered (Additional file 1). The categories used in the Audit Trigger Questions can also be used to prepare a post audit action plan to document actions and responsibilities (Additional file 2). Reviewing the questions can be time consuming and is best conducted separately by participants to ensure a wide range of issues are considered. Each party forwards their priority issues to the facilitator who reviews the issues raised for concordance with the terms of reference and scope of the audit and prepares a final list of issues for discussion and a brief summary of the outbreak to provide context.

For national outbreaks, state-based health agencies may conduct mini-audits among local health agencies in their own jurisdiction and contribute their findings to a national audit. A focus on the perceived "failings" of a single agency or unit should be avoided.

In summary, prior to the audit meeting the following should be accomplished:

• The lead agency proposes the terms of reference and scope of the audit

• The facilitator confirms and/or modifies terms of reference and scope of audit in consultation with the participants.

• All participants/organisations should submit issues for review based on the Audit Trigger Questions (Additional file 1).

• The facilitator reviews issues and compiles a list of discussion points

• The facilitator circulates the list of discussion points, statement of scope, and brief summary of outbreak to audit attendees and other stakeholders.

#### Conducting the Audit Meeting

**Figure 1:**
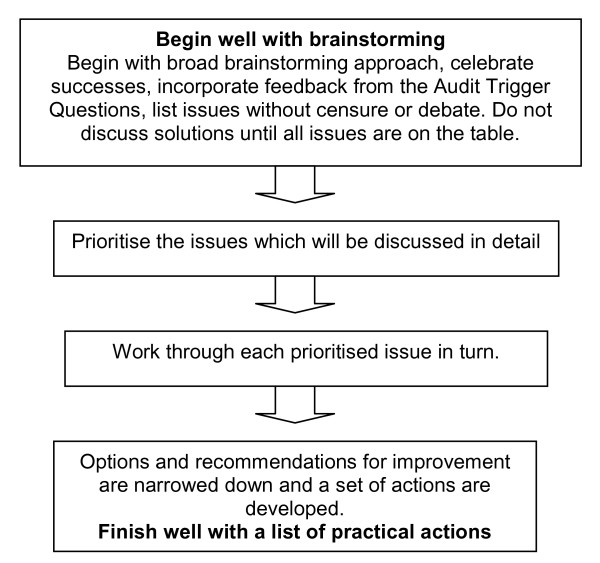
**Conduct of the audit meeting**. Begin with a broad exploration of issues to ensure all views are heard; brainstorming without censure in the middle, and narrowing down to practical actions in the end.

Audits begin with an exploration of issues (Figure 1), during which summary information on the outbreak is shared with the group, along with feedback from the Audit Trigger Questions review (Additional file 1).

This is followed by brainstorming in an open session in which participants offer brief observations, uncensored and unevaluated by self or others, on the conduct of the outbreak without further discussion of the observations - except for questions of clarification. All observations are recorded and only after an agreed period, 10 to 20 minutes perhaps, are they evaluated and prioritised for more interactive discussion. The emphasis is on openness and creativeness. After the issues in the prioritised list are discussed in full, the parties then return to the first prioritised issue and take turns at proposing solutions. The facilitator assists by reframing negative statements into neutral or positive language that reflects the parties interests rather than their positions[10]. An example of reframing a statement to identify interests rather than positions follows:

**Party A**: "Party B should not give advice to the facility, it is our role not theirs."

**Facilitator**: "Can you tell Party B, more about your experience of them giving advice to the facility and it's impact?"

**Party A**: "We gave the facility different advice to Party B and it caused confusion for the facility manager."

**Facilitator**: "So would it be accurate to say that your main interest is to ensure that your agency and Party B do not give conflicting advice to facilities during joint investigations?"

**Party A**: "Yes, that would be accurate."

**Facilitator**: "Party B - would you share the same interest in ensuring facilities were not given conflicting information and clarifying your respective roles in this area?"

Focusing on system issues will often reveal that interpersonal conflict has its root in organisational systems. Occasionally personality conflicts will be elevated to an organisation level. Conflict is facilitated and amplified by differences in organisational culture, legislation, structure and political imperatives between jurisdictions. A lack of clarity of roles, or the failure to clearly articulate and document the respective roles of agencies in outbreak investigations may manifest as interpersonal conflict. Where there is conflict the audit process should not be seen as a method for determining which group was right or wrong or who performed well or poorly. The audit process should be used to reorient damaging communication styles and model effective communication techniques. In this setting a skilled external facilitator may be needed to encourage interest-based negotiation of contested issues in an environment that allows stakeholders to hear and express needs and concerns that were not able to be heard during the urgent and often politically charged environment of an outbreak response[11].

It is important to resist the temptation to rush to proposing solutions until all the issues have been articulated by the stakeholders. All suggestions should be recorded, with particular emphasis on practical solutions. When suggestions are exhausted, the group should prioritise the action items for discussion.

Where performance indicators or guidelines exist, for example in agency outbreak response or disease control manuals, these should be considered during the audit and may be revised following the audit. As best practice has not been formulated for many aspects of outbreak investigation the audit should aim to collect data which can be used to define and describe best practice. This methodology should contribute to more explicit quality standards for outbreak investigation or at least an ability to benchmark against peers in terms of objective measures, for example the timeliness of responses in outbreaks.

### Confidentiality agreement

How reports of the audit will be prepared and their extent of dissemination should be agreed by participants in advance. Confidentiality may be important if participants are to discuss the outbreak response freely. Barriers to best practice should be identified, discussed, and recorded without attributing blame. Records of the audit can be made in such a way as not to identify agencies and personnel unnecessarily. The need for confidentiality must be balanced against the ethical need to disseminate learnings to broader stakeholder groups, and be understood in the context of the relevant freedom of information and privacy legislation. Consideration may be given to the use of the Chatham House Rule[12] in which information derived from the meeting may be discussed in general terms but the source of the information remains confidential.

### Implementation and dissemination of recommendations

The recommendations from the audit should be concrete and able to be implemented. The facilitator should contact the participants after four to six weeks to review progress on the agreed actions, exploring any barriers or difficulties that can be overcome. Additional file 2 provides a template for organising an accountable report of the audit to assist in implementation of the recommendations. Publishing a final report of the findings and recommendations will assist future public health practice improvements locally and dissemination via the web or in the peer-reviewed literature and through presentation at public health grand rounds will enhance learning more broadly.

A brief evaluation following the most recent two structured audits allowed each participant to provide written feedback on the following questions: 1) was the process/methodology clear? 2) did the structured methodology assist or inhibit the debrief? 3) do you have any suggestions for the facilitator to improve facilitation of debriefs in the future? and 4) other general comments.

## Results

Five outbreaks have undergone structured audit between 1997 and 2008, with participants ranging from a single regional health protection unit through to inter-agency and multi-jurisdictional audits. These identified a broad range of outbreak response quality improvement measures at national, state and local level (Table 2).

**Table 2:** Characteristics and key issues identified using the structured audit methodology in Australia.

Outbreak Response	Participants	Key Issues Identified for Improvement*
Multi-state outbreak of Salmonella *Mbandaka *associated with contaminated peanut butter, Australia,1995[7,8]	State and territory communicable disease and laboratory heads.	Need for national coordination of outbreaks and clarification of national and jurisdictional roles. Need for national laboratory network to provide rapid subtyping of isolates. Development of performance standards for timeliness of outbreak response.

Influenza outbreaks in aged-care facilities, New South Wales, 2005 [13].	Regional health protection staff and Federal health department aged-care accreditation staff	Identify a coordinator to facilitate communication between all stakeholders - facilities, state and federal agencies. Develop standard surveillance case definitions, notification triggers, and national guidelines for influenza control in aged-care facilities. Clarify roles of general practitioners in outbreaks. Promotion of influenza immunisation in aged-care staff and residents. Need for broadcast facsimile capacity to aged-care facilities. Need for daily communication with affected families during quarantine.

Prophylaxis of contacts of food handler with hepatitis A to prevent an outbreak, NSW, 2006.**	Regional health protection staff	Promote early notification of hepatitis from laboratories and general practitioners. Need for coordination of communication and liaison with other agencies e.g. schools and food safety authority. Ensure roles of all staff are clear and documented in rapidly evolving outbreak responses. Development of fact sheets and mass vaccination procedures.

Salmonella outbreak in an aged-care facility, NSW, 2008.**	Regional health protection staff and food safety authority	Develop guidelines for appropriate investigation timelines and share them between agencies. Document roles of each agency and agree on and share communicated advice in each outbreak setting.

Salmonella outbreak in a restaurant, NSW, 2008.**	Regional health protection staff and food safety authority	Develop protocols for joint health and food authority investigations with clarification of roles and legal status. Need for rapid sharing of new epidemiological or environmental investigation information. Interagency governance procedures identified for reporting and implementing recommendations from structured outbreak audits.

*Selected issues only. ** Unpublished internal reports Hunter New England Population Health

We trialled qualitatively rating outbreak investigation performance against the Audit Trigger Questions using criteria such as "adequate" or "needs improvement", however, it was found to be cumbersome and slowed down the preparation for the audit and subsequently this was dropped in favour of placing a tick beside those questions that should be addressed in the audit.

Initial results from confidential written evaluations from eight participants from the last two structured audits demonstrate that participants consider the process and methodology to be clear and useful. Some participants find that while the structure of the audit assists and helps to make the process "neutral" and "non-threatening" it may also limit the discussion of issues that arise during the audit because these are considered "out of scope". The circulation of a document summarising the outbreak, the scope of the audit and issues suggested for discussion prior to the audit was considered valuable. It was suggested that where possible the facilitator should be a neutral party and that sometimes limiting the audit to two hours inhibited exploration of issues such as future prevention measures.

## Discussion

The following issues were repeatedly identified across several structured audits: the need for clear definitions of roles and responsibilities both within and between agencies, communication between agencies and with external stakeholders involved in outbreaks, and the need for development of performance standards in outbreak investigations - particularly in relation to timeliness of response.

The methodology used in the audit is acceptable to participants and there is support for continued use and development of the tool. We have found that the focus on common interests of the parties, positive reframing of issues, and compliance with the structure of the audit has minimised the potential for interpersonal conflict during the audit meetings. Agencies welcome guidance in approaching the evaluation of complex outbreak responses and this methodology has been chosen by the Public Health Laboratory Network of Australia to review the laboratory response to the 2009 influenza pandemic.

The methodology for the audit has evolved over time, while it was initially structured around the Audit Trigger Questions in Additional file 1, the process has been increasingly informed by mediation or alternative dispute resolution principles which assist in the open exploration of issues in a non-judgemental environment and prevents premature decisions on solutions before underlying systems issues are fully explored[11].

The importance of a supportive environment for structured audit cannot be over emphasised. In clinical audit, the most frequently cited barrier to successful clinical audit is the failure of organisations to provide sufficient protected time for healthcare teams and it likely the same would apply to public health agencies[1]. In our experience, as in clinical audit, there is a need for an organisational commitment to implement the recommendations that flow from an audit as the good intentions of the audit participants may not be sufficient for organisational change. Organisations may benefit from formally incorporating audit reports into an established reporting process for quality improvement or risk management[1]. This may require reporting audit outcomes and organisational commitments to a higher executive level to promote accountability, a practice recently adopted in the local audits conducted.

Kipping et al highlighted the lack of published standards for auditing outbreak response and emphasised that further development and promotion of such standards was required[6].

This methodology is evolving with practice and we encourage feedback and modification of the process from practitioners. Future challenges include incorporating feedback from broader stakeholder groups, for example those of affected cases, institutions and businesses; assessing the quality of audit; developing training for both participants and facilitators; and building a central capacity to support jurisdictions conducting audits. The incorporation of measurable performance criteria or sharing of benchmarkable performance criteria will assist in the standardisation of audit and further quality improvement. A Google Group has been initiated at http://groups.google.com/group/outbreak-audits to promote use of the methodology and the development of a collaborative network to share learning and modifications of the structured audit methodology.

## Conclusion

The framework was acceptable to participants, provided an opportunity for clarifying perceptions and enhancing partnership approaches, and provided useful recommendations for approaching future outbreaks. Future challenges include incorporating feedback from broader stakeholder groups, for example those of affected cases, institutions and businesses; assessing the quality of a specific audit; developing training for both participants and facilitators; and building a central capacity to support jurisdictions embarking on an audit. The incorporation of measurable performance criteria or sharing of benchmark performance criteria will assist in the standardisation of outbreak investigation audit and further quality improvement.

## Competing interests

The authors declare that they have no competing interests.

## Authors' contributions

CBD developed the initial drafts of the Audit Trigger Questions and criteria for auditing outbreaks, introduced the alternative dispute resolution principles to the audit and drafted the initial manuscript. TDM and SAM developed the template for reporting the outcome of the audits and further refined the audit process. DND and MDK provided intellectual input into design and conduct of the audit. All authors helped draft and revise the manuscript.

## Pre-publication history

The pre-publication history for this paper can be accessed here:

http://www.biomedcentral.com/1471-2458/9/472/prepub

## Supplementary Material

Additional file 1 **Appendix 1 - Audit Trigger Questions**. Tabular checklist of questions to trigger further exploration in the structured audit. Click here for file

Additional file 2 **Appendix 2 - Post-audit Action Plan Template**. Tabular template for recording post-audit actions and recommendations. Click here for file
