# Supplementary material for: A structured framework for improving outbreak investigation audits
Source: BMC Public Health. 2009 Dec 18;9:472. doi: 10.1186/1471-2458-9-472 (PMC2813237; doi:10.1186/1471-2458-9-472)
Supplement: Additional file 1 — Appendix 1 - Audit Trigger Questions. Tabular checklist of questions to trigger further exploration in the structured audit. [file 1471-2458-9-472-S1.DOC]

Additional File 1

Audit Trigger Questions

The questions below are not intended to be addressed in their entirety during a structured audit but should be used to prompt stakeholders to nominate a wide range of issues for consideration during the structured audit.

Prevention/mitigation

|  | **Issue** | **Tick for inclusion in audit** |
| --- | --- | --- |
| 1 | Does public health legislation allow collection of appropriate surveillance data? | ❑ |
| 2 | How often is surveillance data audited by an epidemiologist? | ❑ |
| 3 | Is a log kept of the epidemiologist’s interpretation of the data? | ❑ |
| 4 | Does regular analysis of surveillance data allow early detection of outbreaks? | ❑ |
| 5 | Are regional, national and international trends in communicable diseases monitored to allow prediction of emerging threats within the agencies’ jurisdiction? | ❑ |
| 6 | Do adequate programs exist to prevent outbreaks of this disease? | ❑ |

Preparedness

|  | **Issue** | **Tick for inclusion in audit** |
| --- | --- | --- |
| 1 | Do public health agencies have adequate numbers of trained staff to respond to outbreaks? | ❑ |
| 2 | Do staff have formal epidemiological qualifications/training? | ❑ |
| 3 | Do staff attend/run refresher courses? | ❑ |
| 4 | Are structured audits of response to (appropriate) outbreaks conducted? | ❑ |
| 5 | Does the agency have guidelines or performance standards for outbreak response? | ❑ |
| 6 | Does the agency have access to additional personnel and laboratory resources for response to outbreaks? | ❑ |
| 7 | Does the agency have predefined financial resources to allocate to outbreak control and investigation? | ❑ |
| 8 | Was the response restrained by financial or other resources? | ❑ |
| 9 | Does the agency have pre-prepared sources of public information (brochures/web-based) for dissemination in the event of an outbreak? | ❑ |
| 10 | Are there clear organisational, coordination, and communication structures that define jurisdictions’ responsibilities established at the local, state and national level? | ❑ |
| 11 | Can the agency establish and staff a telephone information hotline/recorded information line/website rapidly (<24 hours) following identification of an outbreak? | ❑ |

Response

|  | **Issue** | **Tick for inclusion in audit** |
| --- | --- | --- |
| Epidemiologic Investigation | |  |
| 1 | Was the outbreak detected by any formal surveillance system? | ❑ |
| 2 | Were surveillance data from other states/regions/countries reviewed for increases? | ❑ |
| 3 | Were unreported cases sought? | ❑ |
| 4 | Was the outbreak recognised in time to investigate the cause? | ❑ |
| 5 | Was a standard case definition developed and disseminated to all stakeholders? | ❑ |
| 6 | How soon after recognition of the outbreak were hypothesis-generating interviews conducted? | ❑ |
| 7 | Could a case-control or cohort study have been conducted to identify the cause or source of infection? | ❑ |
| 8 | If the outbreak involved a number of states or regions, was a standard data collection instrument used both within and between states? | ❑ |
| 9 | Was the method of contacting exposed or potentially exposed people appropriate? | ❑ |
| 10 | Were general practitioners offered the opportunity to make initial contact with their patients ? | ❑ |
| 11 | How were people contacted by phone, letter or other (specify)? | ❑ |
| 12 | How long did it take to contact cases or contacts? | ❑ |
| 13 | Was simple, clearly written information made available at the time of first contact? | ❑ |
| 14 | Were arrangements made for laboratory testing at the time of initial contact? | ❑ |
| 15 | Was frequent contact maintained with infected people to ensure that they received appropriate support or counselling? | ❑ |
| Contact Management | |  |
| 1 | Was a definition of a “contact” established and communicated to all stakeholders? | ❑ |
| 2 | Were contacts given written information on their risks, early symptoms of disease, isolation or quarantine and triggers for contacting the health department? | ❑ |
| 3 | Were adequate prophylactic medications available with protocols for storage and distribution? | ❑ |
| *Environmental Investigation* | |  |
| 1 | If a contaminated vehicle or environment was suspected, how long did it take to obtain specimens from the suspected vehicle or environment? | ❑ |
| 2 | How long did it take to obtain product distribution/passenger/ patron lists? | ❑ |
| 3 | How long did it take to withdraw the contaminated product from circulation? | ❑ |
| 4 | How long before the production facility was inspected? | ❑ |
| 5 | How long before the appropriate regulatory body was notified? | ❑ |
| 6 | How was the success of product recall verified? | ❑ |
| 7 | Was staff safety ensured during the outbreak response? | ❑ |
| 8 | Was industry/private stakeholder assistance sought? | ❑ |
| *Laboratory Investigation* | |  |
| 1 | What proportion of people affected by the outbreak were asked for specimens? | ❑ |
| 2 | What proportion gave specimens? | ❑ |
| 3 | What specimens were collected? | ❑ |
| 4 | Were the samples adequately labelled, transported (consider personal delivery) and stored? | ❑ |
| 5 | Were appropriate laboratory tests available for rapid diagnosis? | ❑ |
| 6 | If not, were funds available for the rapid development of diagnostic tests? | ❑ |
| 7 | For readily available testing, were standard procedures used by all laboratories? | ❑ |
| 8 | How quickly was a laboratory found that could do all appropriate testing? | ❑ |
| 9 | How long did it take for all testing to be completed? | ❑ |
| 10 | How long did it take for subtyping of isolates? | ❑ |
| 11 | Were autopsies conducted on people who died as a result of the infection? | ❑ |
| *Communication* | |  |
| 1 | How quickly was the national agency notified of an outbreak of national significance? | ❑ |
| 2 | Were other relevant agencies contacted and if so, when? | ❑ |
| 3 | How long after recognition of the outbreak were informational materials identified or developed? | ❑ |
| 4 | Was a hotline/website set up to provide information to the community? | ❑ |
| 5 | Were hospitals, emergency departments, laboratories and medical practitioners provided with appropriate and timely information? | ❑ |
| 6 | Was there a coordinated response to the media - one person identified to coordinate response, regularly scheduled press conferences? | ❑ |
| 7 | Were public health preventive messages clearly defined? | ❑ |
| 8 | Was success of public relations activities evaluated? | ❑ |
| 9 | Were epidemiological, environmental, and laboratory findings obtained in different jurisdictions shared with other relevant health related agencies? | ❑ |
| 10 | Were records of all telephone calls, meeting minutes, and major decisions logged contemporaneously? | ❑ |
| 11 | Were meetings with a comprehensive agenda established on a daily or frequent basis within the investigation team and with external stakeholders? | ❑ |
| 12 | Were national or state incident protocols activated? | ❑ |
| 13 | Were regular updates provided to affected community, industry and other stakeholders and how was the dissemination of this information coordinated? | ❑ |
| 14 | Were non-government stakeholders provided with a written description of the roles of (perhaps multiple) agencies in outbreak response? | ❑ |
| 15 | Were managers of affected facilities provided with disease control advice in writing? | ❑ |
| *Public Health Action* | |  |
| 1 | Was the outbreak identified in a sufficiently timely fashion to prevent ongoing risk of disease? | ❑ |
| 2 | How long before recall of a product or cessation of a risk activity? | ❑ |
| 3 | Could this have happened more quickly and if so, what were the impediments? | ❑ |
| 4 | Was counselling provided to confirmed cases and families? | ❑ |
| *Outbreak Management* | |  |
| 1 | Were all role holders clear on their own roles and those of others in the team and other agencies? | ❑ |
| 2 | Was the mix of staff numbers and skill mix adequate? | ❑ |
| 3 | Was any further equipment required? | ❑ |
| 4 | Were ethical, legal, and privacy issues adequately considered and addressed? | ❑ |
| 5 | Were the facilities used appropriate for the task? | ❑ |
| 6 | Was distance or travel an impediment to the investigation? | ❑ |

Recovery

|  | **Issue** | **Tick for inclusion in audit** |
| --- | --- | --- |
| 1 | Was there a structured debriefing at the agency or agencies involved? | ❑ |
| 2 | Was counselling for cases, agency staff, or other stakeholders provided, as necessary? | ❑ |
| 3 | Were reasons for the outbreak and risk factors for infection identified and published? | ❑ |
| 4 | Was a report on the outbreak provided to the community, data providers, colleagues and wider public health community? How and when was the information communicated? | ❑ |
| 5 | Was the need for further studies identified (eg epidemiologic, laboratory or economic impact studies)? | ❑ |
| 6 | Was the adequacy of surveillance systems reviewed? Were weaknesses identified and rectified? | ❑ |
| 7 | Was there an evaluation of the impact of the public health intervention (eg number of secondary cases prevented)? | ❑ |
| 8 | Is there now a program in place that could prevent another outbreak? | ❑ |
| 9 | Were the outbreak control guidelines reviewed? | ❑ |
| 10 | Does the organisation provide protected time for staff to invest in outbreak investigation audits? | ❑ |
| 11 | Is there a clear method for disseminating and implementing recommendations from audits of outbreak investigations in the organisation? | ❑ |
