# Supplementary material for: A structured framework for improving outbreak investigation audits
Source: BMC Public Health. 2009 Dec 18;9:472. doi: 10.1186/1471-2458-9-472 (PMC2813237; doi:10.1186/1471-2458-9-472)
Supplement: Additional file 2 — Appendix 2 - Post-audit Action Plan Template. Tabular template for recording post-audit actions and recommendations. [file 1471-2458-9-472-S2.DOC]

# Additional File 2

# Post-audit Action Template

Each section below includes examples of possible topics for discussion in italics. The audit trigger questions (Additional File 1) include a full list of topics for consideration. Record actions in this template at the end of the audit.

| Topic | Issues identified | Action required | By whom | Date due |
| --- | --- | --- | --- | --- |
| **PREVENTION of future outbreaks** |  |  |  |  |
| *eg. Programs in place to prevent a future outbreak?* |  |  |  |  |
|  |  |  |  |  |
| **PREPAREDNESS for this outbreak** |  |  |  |  |
| *eg. Clear jurisdictional responsibilities and structures for cross-jurisdictional communication and coordination?* |  |  |  |  |
| *eg. Appropriate staff numbers and skills?* |  |  |  |  |
|  |  |  |  |  |
| **RESPONSE to this outbreak** |  |  |  |  |
| **Outbreak detection**  *eg. Timeliness?* |  |  |  |  |
| **Investigation**  *eg. Appropriateness of investigation type?*  *eg. Timeliness of investigation?* |  |  |  |  |
| **Contact management**  *e.g. Definition of contact?*  *e.g. Provision of information to contacts?* |  |  |  |  |
| **Environmental Investigation**  *e.g. Timeliness of sample collections and controls?* |  |  |  |  |
| **Laboratory issues**  *eg. Specimen collection and handling?*  *eg. Timeliness of specimen transport and testing?* |  |  |  |  |
| **Response logistics and resources**  *eg. Coordination of outbreak?*  *eg. Adequate staff numbers and skill mix?* |  |  |  |  |
| **Communication**  *eg. Within response team?*  *eg. With State and National bodies?*  *eg. With GPs, EDs, Labs?*  *eg. Which stakeholders were contacted?*  *eg. Timeliness of referral?* |  |  |  |  |
| **Outbreak management**  *e.g. Staff roles clear?*  *e.g. Staff mix and number?*  *eg. Staff safety?*  *eg. Legal issues?* |  |  |  |  |
| **Other issues** |  |  |  |  |
|  |  |  |  |  |
| **RECOVERY from this outbreak** |  |  |  |  |
| *eg. Final report prepared?*  *eg. Report for community?* |  |  |  |  |
|  |  |  |  |  |
